# Hafnium Oxide-Based Nanostructures as Powders and in Polyvinyl Alcohol Hydrogels for Light-Assisted Processes

**DOI:** 10.3390/gels12050405

**Published:** 2026-05-08

**Authors:** Mihai Anastasescu, Polona Umek, Cristina Maria Vladut, Veronica Bratan, Catalin Negrila, Silviu Preda, Luminita Predoana, Catalina Gifu, Cristina Lavinia Nistor, Daniela C. Culita, Daiana Mitrea, Crina Anastasescu, Maria Zaharescu, Ioan Balint

**Affiliations:** 1 Institute of Physical Chemistry “Ilie Murgulescu”, 202 Splaiul Independenţei, 060021 Bucharest, Romania; manastasescu@icf.ro (M.A.); vladut@icf.ro (C.M.V.); vbratan@icf.ro (V.B.); predas@icf.ro (S.P.); lpredoana@icf.ro (L.P.); danaculita@yahoo.co.uk (D.C.C.); daiana.mitrea@yahoo.com (D.M.); mzaharescu@icf.ro (M.Z.); 2 Jožef Stefan Institute, Jamova Cesta 39, SI-1000 Ljubljana, Slovenia; polona.umek@ijs.si; 3 National Institute of Materials Physics, 405A Atomiştilor, P.O. Box MG 7, 077125 Măgurele, Romania; catalin.negrila@infim.ro; 4 National Research and Development Institute for Chemistry and Petrochemistry—ICECHIM, 202 Splaiul Independentei, 060021 Bucharest, Romania; catalina.gifu@icechim-pd.ro (C.G.); cristina.nistor@icechim-pd.ro (C.L.N.)

**Keywords:** hafnium oxide nanostructures, PVA hydrogels, films, powders, reactive oxygen species, protoporphyrin IX, quenching of DL α-tocopherol, simulated/visible solar light, photocatalytic activity

## Abstract

Hafnia (hafnium oxide) nanostructures, both unmodified and silica-modified with minor and major silica content, were synthesized using an adapted sol–gel method with D-L tartaric acid as an internal template. After thermal treatment, structural non-stoichiometry and light absorptive properties were identified in the resulting hafnium-based nanostructures, indicating their potential for various applications, including photocatalysis. The ability of these materials to photogenerate reactive oxygen species (ROS), namely superoxide anion radicals (•O_2−_) under simulated solar light (AM 1.5) and singlet oxygen (^1^O_2_) under visible light (λ > 390 nm), was evaluated and monitored by UV–Vis and photoluminescence spectroscopy. Functionalization of hafnium-based oxides with protoporphyrin IX was employed to enhance singlet oxygen photogeneration. The reactivity of the generated (^1^O_2_) was assessed by quenching of DL α-tocopherol photoluminescence under visible light irradiation. Photocatalytic experiments conducted under anaerobic conditions demonstrated the ability of the hafnia-based nanostructures to reduce 1,4-benzoquinone (BQ) to 1,4-hydroquinone (H_2_Q). Furthermore, embedding the hafnia-based powders into polyvinyl alcohol hydrogels enabled the obtainment of photoactive coatings on glass substrates, for which their mechanical properties were evaluated using force–distance spectroscopy measurements. Morphological and structural characterization of the materials was performed using scanning electron microscopy (SEM), scanning transmission electron microscopy (STEM), atomic force microscopy (AFM), X-ray diffraction and fluorescence (XRD, XRF), X-ray photoelectron spectroscopy (XPS), N_2_ adsorption–desorption measurements, UV–Vis spectroscopy, photoluminescence (PL) spectroscopy, and zeta potential measurements. These investigations revealed that adding silica induces significant modifications in the morphology, texture, and structure of the hafnia, thereby enhancing the functional properties of the resulting materials.

## 1. Introduction

The development of new engineered materials, as well as the enhancement of conventional ones through the use of modifiers and advanced processing strategies, together with their integration with renewable and clean energy sources such as solar light, has become imperative in current research. Accordingly, this work presents new approaches for the development of photoactive hafnium oxide-based powders under simulated solar light exposure. Their incorporation into PVA hydrogels and subsequent processing as films on glass substrates are intended to expand the potential applications of the resulting photoactive systems, particularly in the biomedical field. Hafnium oxide is mainly known as a “high-k dielectric material” that is extensively used in microelectronics to replace SiO_2_ in silicon-based devices, as well as in optical coatings and optoelectronics. Its wide range of applications is due to its various properties, including high thermal, mechanical, and chemical stability; high dielectric permittivity (k = 16–25); a large band gap energy (Eg = 5.4–5.6 eV); and a high refractive index [1,2,3]. HfO_2_ exists in cubic, tetragonal, and monoclinic crystalline phases, with the monoclinic phase being the most common and thermodynamically stable under standard conditions [4,5]. Its properties depend on nanoparticle size and morphology [6,7], which can be tailored through various synthesis methods, such as sol–gel [8], hydrothermal synthesis, chemical vapor deposition (CVD) [9,10], and co-precipitation [11]. According to Gritsenko et al. [1], hafnia contains oxygen vacancies as predominant defects that act as electron and hole traps, promoting the electronic transitions between defect-induced states and modulating the material’s light absorption capacity, luminescence, and charge transport properties [12]. Therefore, their presence and structure have been intensively investigated using various approaches, including spectroscopic analyses and charge transport properties [13,14,15,16]. By employing modifiers, different post-synthesis treatments, or alternative processing approaches such as film deposition for hafnia-based materials [17], the type and density of defects, as well as the electronic structure, can be tuned [18], enabling the extension of their applications towards catalysis and photo-/electrocatalysis fields that have been less explored so far. One example is hydrogen photogeneration via water splitting under UV irradiation using HfO_2_ nanorods films [19]. In general, research efforts have focused on narrowing the band gap of HfO_2_ in order to shift its photoactivity from the UV to the solar/visible region. This has been achieved through various modification strategies, resulting in Mn-modified HfO_2_ nanoparticles [4], cerium-modified HfO_2_ [20], deposition of hafnium oxide nanoparticles on graphene nanosheets [21], and Pt/Ru nanoparticle-decorated hafnium oxide nanohybrids for the visible light-driven photocatalytic removal of antibiotics [22]. Therefore, it can be assumed that the use of hafnia-based materials in photocatalysis remains an open research topic that has not yet been fully investigated or established. Our previous studies on the photocatalytic activity of amorphous SiO_2_ with tubular morphology and optically active defects demonstrated that synthesis optimization aimed at increasing the defect density on the nanotube surface can promote photocatalytic processes, such as methanol photodegradation in aqueous media, even at the surface of an insulating material. This approach, based on nonstoichiometric insulators enriched with defects and capable of triggering photocatalytic processes, has also been highlighted by Li et al. [23].

Herein, hafnium oxide nanostructures were synthesized using a method similar to that employed for SiO_2_ nanotubes (i.e., an adapted sol–gel approach), with the aim of generating optically active defects. Additionally, the materials were modified with SiO_2_ to create new active interfaces. The present work represents a preliminary study toward future photocatalytic applications, investigating the ability of the HfO_2_ to transfer photogenerated electrons from its surface to benzoquinone (BQ), thereby triggering its reduction to hydroquinone (H_2_Q), for which its formation was monitored spectrophotometrically. In addition, to emphasize the ability of the hafnium-based materials to generate electrons at their surface under simulated solar irradiation, the formation of the superoxide anion radicals (•O_2−_) was also monitored. On the other hand, hafnium dioxide (HfO_2_) was used for biomedical applications due to its high density, corrosion resistance, and cytocompatibility [24,25], being involved in prosthetics and controlled drug delivery systems. Recent studies have investigated the potential use of modified HfO_2_ nanoparticles as theranostic agents, owing to their ability to accumulate in cancer cells and their suitable properties for supporting photodynamic and radiotherapy treatments [26,27], which primarily rely on the generation of reactive oxygen species (ROS) under different types of irradiation. The use of inorganic nanomaterials as nano-photosensitizers in photodynamic therapy (PDT) has been extensively investigated in recent years [28], as has the involvement of singlet oxygen in light-assisted therapies and environmental remediation processes [29].

This work also includes structural and functional investigations of the HfO_2_-based materials under light irradiation, aiming to monitor singlet oxygen generation (^1^O_2_) and its reactivity as a prerequisite for the potential application of these materials in photodynamic therapy (PDT), both as simple powders and when embedded in appropriate, transparent gels for potential clinical applications. Based on our previous studies on amorphous SiO_2_ with tubular morphology, light-absorbing defects, photocatalytic activity, and singlet oxygen providers [30,31], hafnium oxide has been similarly obtained and modified with SiO_2_ in order to generate singlet oxygen under light exposure. In the case of SiO_2_, the addition of inorganic (metal NPs) and organic (ruthenium dye) sensitizers was shown to enhance the light absorption capacity of the resulting hybrid materials, as well as their ability to generate singlet oxygen under solar and visible light irradiation, leading to antibacterial activity against *Staphylococcus aureus* [32]. The embedding of these materials in PVA hydrogels preserved their structural and functional properties. Furthermore, porphyrin immobilization on SiO_2_ improved singlet oxygen generation and its reactivity toward DL α-tocopherol under solar/visible light irradiation [33]. Therefore, in this study, hybrid derivatives of hafnium-based oxides with protoporhyrin IX (denoted as PHfO_2_, PHfO_2_SiO_2_ I, PHfO_2_SiO_2_ II) were developed and evaluated for singlet oxygen generation and photoluminescence (PL) quenching of DL α-tocopherol under simulated solar irradiation. These investigations were performed on both powders and films (deposited on glass) containing PVA hydrogels embedded in the inorganic and porphyrin-derived hybrid powders. The development of HfO_2_-based materials, including both inorganic and hybrid systems (obtained by immobilizing protoporphyrin IX on the investigated inorganic carriers), as singlet oxygen generators under solar/visible light irradiation is aimed at future potential applications in the biomaterials and biomedical field. In this context, embedding porphyrin-derived hybrid powders into PVA hydrogels as singlet oxygen generators could represent an initial step toward the development of photobioactive gels for dental treatments or cancer therapy.

The aim of this paper is (a) to firstly develop and extensively characterize (structural and functional) optically active advanced materials (powders) based on HfO_2_ modified with SiO_2_ in order to extend the conventional application domain of hafnia (micro- and optoelectronics) towards photocatalytic and biomedical applications; (b) subsequently, we embed the abovementioned powders in PVA hydrogels and deposit them on glass as photo-/bioactive films. These films could be extensively used for air depollution processes and also as bioactive films/coatings with potential antibacterial activity. These application areas are interconnected in this work through the common aspect of reactive oxygen species (ROS) photogeneration.

## 2. Results and Discussion

### 2.1. Scanning Electron Microscopy (SEM)

**Figure 1:**
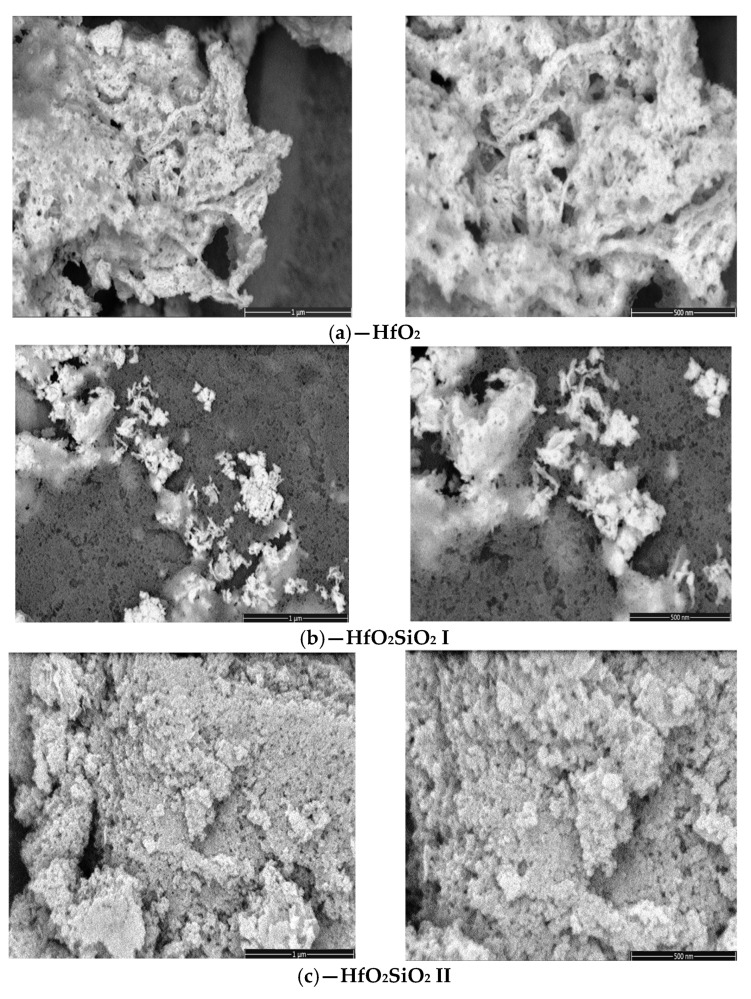
SEM images of HfO_2_ (**a**), HfO_2_SiO_2_ I (**b**), and HfO_2_SiO_2_ II (**c**) powders.

Figure 1 presents the SEM images of the HfO_2_, HfO_2_SiO_2_ I, and HfO_2_SiO_2_ II samples, revealing fine and homogeneous nanostructures for all materials. In the case of pure HfO_2_ (Figure 1a), fibrous structures forming a porous network can be observed. The morphological characteristics of the HfO_2_SiO_2_ I sample (Figure 1b) are quite similar to those of the first sample (containing only 0.68 wt% Si relative to Hf), but with a thinner network of nanofibrils and additional inhomogeneities, likely due to the presence of SiO_2_. The third sample, namely HfO_2_SiO_2_ II (Figure 1c), exhibits a denser aggregation of nanoparticles compared to the first sample (Figure 1a). Its morphology appears distinctly different, being predominantly composed of SiO_2_, with HfO_2_ present as a minor phase.

**Figure 2:**
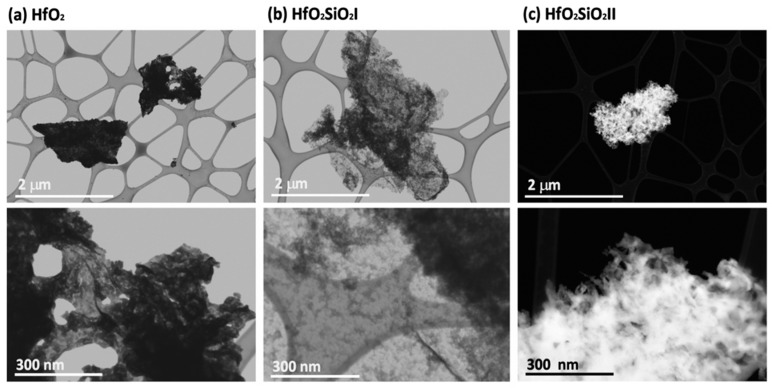
STEM-in-SEM images of HfO_2_ (**a**), HfO_2_SiO_2_ I (**b**), and HfO_2_SiO_2_ II (**c**). Images were taken in BF (**a**,**b**) and HAADF (**c**) modes.

Figure 2 reveals the characteristic cluster size for all three synthesized powders. The cluster size, in the case of HfO_2_ and HfO_2_SiO_2_ II, is around 2 μm, while clusters of HfO_2_SiO_2_ I appear to be bigger, up to 3 μm. HAADF images (Figure 2c) suggest that HfO_2_ is relatively well dispersed within the SiO_2_ matrix, as no distinct Hf-rich regions are apparent within the cluster.

### 2.2. Atomic Force Microscopy (AFM)

**Figure 3:**
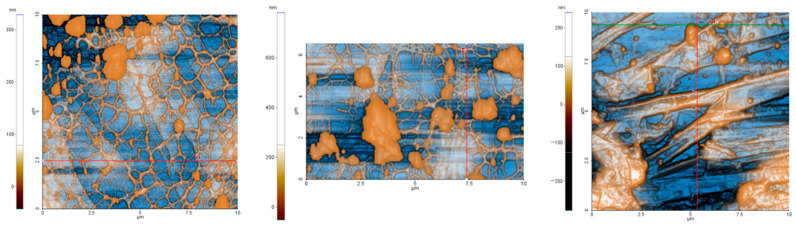

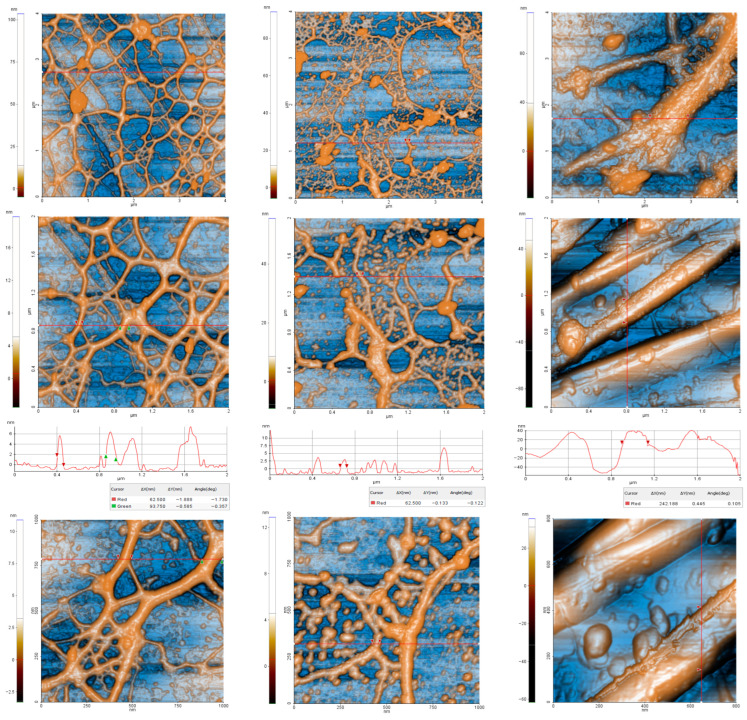
2D AFM images, presented in enhanced contrast mode and acquired at different scan sizes (10 µm—first row; 4 µm—second row; 2 µm—third row; 1 µm—last row) for the HfO_2_ (**left column**), HfO_2_SiO_2_ I (**middle column**), and HfO_2_SiO_2_ II (**right column**) samples. Random line scans were collected from the images scanned at 2 µm × 2 µm and are plotted below the corresponding AFM micrographs.

**Figure 4:**
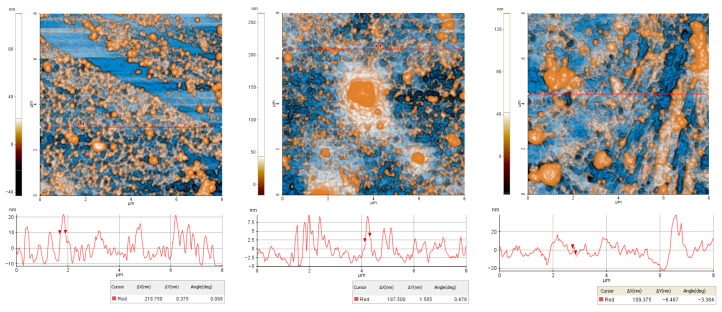

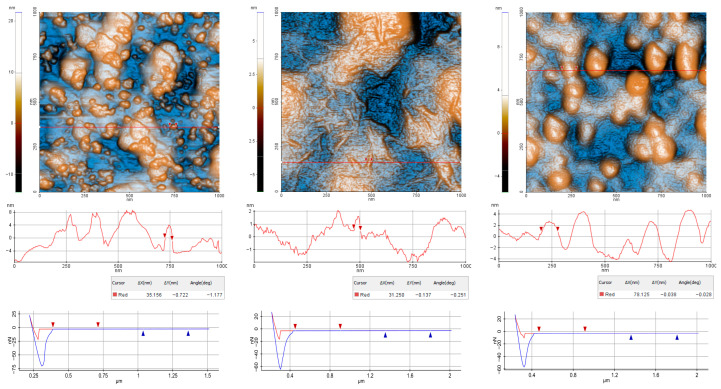
2D AFM images, presented in enhanced contrast mode, acquired over an area of 8 µm × 8 µm for HfO_2_ (**left**), HfO_2_SiO_2_ I (**middle**), and HfO_2_SiO_2_ II (**right**) powders embedded in gels. Higher-resolution AFM images were also recorded over a smaller area of 1 µm × 1 µm, along with random line scans plotted at the position indicated in each image by a red horizontal line. The last row presents the force–distance spectroscopy curves for these gel samples.

AFM was employed to obtain higher-resolution micrographs providing a detailed view of the powders (Figure 3) previously identified by SEM (Figure 1) and of the resulting films formed by incorporating the powders into PVA hydrogels and depositing them on glass substrates (Figure 4). Figure 3 presents the surface topography of the HfO_2_ (left column), HfO_2_SiO_2_ I (middle column) and HfO_2_SiO_2_ II (right column) samples based on 2D AFM images acquired over different scan areas. Characteristic surface profiles (line scans) are shown below the AFM images recorded at a scale of 2 µm × 2 µm, highlighting the dimensions of the selected features (between the two red arrows).

It can be observed that the HfO_2_ (left column) and HfO_2_SiO_2_ I (middle column) samples exhibit a predominant morphology consisting of fibrils and randomly distributed quasi-spherical particles (with a higher number of spherical particles visible in the HfO_2_SiO_2_ I sample, most likely attributable to the presence of SiO_2_). In addition, some aggregated clusters forming hill-like features can be observed, most likely due to insufficient dispersion of the powder. From the images recorded at scan sizes ranging from 4 µm × 4 µm down to 1 µm × 1 µm, it is evident that the HfO_2_ (left) and HfO_2_SiO_2_ I (middle) samples exhibit morphologies composed of interconnected fibrils with diameters starting from tens of nanometers. In contrast, the HfO_2_SiO_2_ II sample (right) displays predominantly rectangular features, with dimensions exceeding 200 nm, in agreement with our previous papers [30,31].

Furthermore, the Hf-based powders were embedded in gels, and Figure 4 presents the surface topography of the resulting dry gels, deposited on glass, containing HfO_2_ (left), HfO_2_SiO_2_ I (middle), and HfO_2_SiO_2_II (right), recorded at scan sizes of 8 µm × 8 µm and 1 µm × 1 µm. As shown in Figure 4, the Hf-based materials are generally well distributed within the gel matrix, with some particles protruding at the surface. These features originate from the embedded powders and exhibit variations in morphology and degree of homogeneity. Roughness parameters were determined for the gel coatings. At a scan size of 8 µm × 8 µm, the HfO_2_ sample exhibits a root mean square roughness (Rq) of 10.9 nm and a peak-to-valley height (Rpv) of 152.7 nm. The HfO_2_SiO_2_ I sample shows higher roughness values (Rq = 21.0 nm, Rpv = 281.2 nm), while the HfO_2_SiO_2_ II sample presents intermediate values (Rq = 19.4 nm, Rpv = 170.8 nm). At a smaller scan size of 1 µm × 1 µm, the following corrugation parameters were obtained: Rq = 5.0 nm and Rpv = 35.2 nm for HfO_2_; Rq = 1.8 nm and Rpv = 13.4 nm for HfO_2_SiO_2_ I; and Rq = 1.7 nm and Rpv = 16.6 nm for HfO_2_SiO_2_ II. Nevertheless, the peculiar morphology of the Hf-based powders remains distinguishable at the surface of the gels. For example, in Figure 4 (second row, middle image), small fibers with diameters of a few tens of nanometers are visible on the surface of the HfO_2_SiO_2_ I-gel-coated glass sample. The nano-mechanical properties of the gels containing the Hf-based materials were tested using force–distance spectroscopy curves (as exemplified in the last row of Figure 3). In these plots, the vertical axis represents the force (expressed in nN), while the horizontal axis corresponds to the tip displacement (µm), with the red curves indicating the approach and the blue curves the retraction process. The contact point is observed around 0.25–0.30 µm, where the force begins to increase sharply towards positive values. The minimum force values (in the range of −55/−75 nN) correspond to the maximum adhesion force required to detach the tip from the sample, indicating significant attractive interactions between the probe and the material. The area between the approach (red) and retraction (blue) curves reflects the energy dissipation during the contact cycle. In force–distance spectroscopy experiments, a tip with a calibrated elastic constant of 0.95 N/m (chosen to balance the deformation of both the sample and the probe) and a radius of curvature of 10 nm was used. Considering a maximum applied force of 21.92 nN (as determined from the recorded force–distance curve), the corresponding cantilever deflection was found to be 23.07 nm. The effective contact was estimated from the graph to ~100 nm so that an indentation of ~76.93 nm was derived. With this data, the indentation energy was obtained as 6.75 × 10^−16^ J, which represents the energy pushed by the tip into the gel film. From the F/d graph, by estimating the retraction area (blue curve), the adhesion energy was computed as 30 × 10^−16^ J, and the total energy dissipated as 33.37 × 10^−16^ J so that the adhesion energy is dominant (>80% of the total energy is lost through detachment, which is specific for polymers). Firstly, using a Hertz model (which provides an apparent Young’s modulus as an approximation) with F = 4/3 E* √R h^(3/2)^ (F—indentation force; R—tip radius; h—penetration depth; E*—effective Young modulus), an effective Young’s modulus of approximately 8.24 MPa was obtained for the HfO_2_SiO_2_ I sample. Applying a correction factor of 0.5 for the Poisson ratio yields a value of ~6.18 MPa. A modulus value of ~8 MPa was reported to be characteristic of soft materials such as polymers or relatively stiff (e.g., dry) hydrogels [34]. Since the contact area between the tip and the sample (~76.9 nm) is much larger than the tip radius (10 nm), this explains the relatively high pull-off force observed, suggesting that the tip may become partially coated with the soft material and resulting in a “molecular suction cup” effect, so additional models can be considered. However, due to the fact that PVA hydrogels exhibit viscoelasticity, combining elastic behavior (storing energy) with viscous behavior (dissipating energy) [35,36], the standard linear solid (SLS/Zener) model was further used, as it can predict both stress relaxation and slow flow (creep) [37,38]. The parameters that can be estimated using the SLS model are the instantaneous modulus (E_0_), which represents the stiffness of the material at initial contact (short term); the equilibrium modulus (E_∞_), related to the stiffness of the material after viscous processes have relaxed (long term); and viscosity (η), which expresses the resistance to flow. The difference between the instantaneous and equilibrium modulus provides information about the viscosity of the material. Besides the information used in the Hertz model (force constant, indentation, and the real force limit) and taking into account the forward speed and forward period, the following viscoelastic parameters were obtained: E_0_ = 8.65 MPa, E_∞_ = 4.94 MPa, and η = 0.371 MPa/s (viscosity). The relatively small value of 8.65 MPa for the instantaneous modulus (E_0_) for the PVA sample (considered a “dry” film) suggests that the evaluated sample contains a residual amount of water. It can be said that the PVA film is not perfectly elastic, since the initial value of 6.18 MPa obtained initially by the Hertz model was an average number between its real stiffness (8.65 MPa) and its relaxed state (4.94 MPa), explaining why the retraction curve (blue) does not overlap the compression curve (red). It can be concluded that the mechanical evaluation of the PVA film by AFM reveals pronounced viscoelastic behavior, with an instantaneous Young’s modulus of 8.65 MPa that relaxes to an equilibrium value of 4.94 MPa. This decrease in stiffness under load, combined with a total dissipated energy of approximately 33.37 × 10^−16^ J, demonstrates the high absorption capacity of the polymer. The values obtained are significantly below the standard for pure, dry PVA (which ranges between 1.5 and 3.0 GPa), suggesting the presence of residual moisture (acting as a plasticizing agent). This result aligns with the literature data for hydrated or hydrogel-like systems [39,40,41,42], where the elastic modulus drops drastically, often below the 10 MPa threshold (e.g., hydrated PVA films reported at ~6.9 MPa or complex hydrogels between 0.4 and 8 MPa). This hydration state favors not only rapid relaxation of the polymer chains but also a dominant adhesion energy, as humidity facilitates the formation of hydrogen bonds and increases the effective contact area during the indentation period. Note that the AFM data, both imagistic and F/d spectroscopy, were obtained on dry gels for the purpose of basic structural characterization and cannot be linked to their in vivo performance. Although the ambient humidity was low (~40% RH), the values obtained (located in the MPa range compared to the theoretical GPa of glassy PVA) confirm the extreme sensitivity of the polymer to water adsorption, explaining the transformation of the film from a rigid solid into an intermediate state, which is characterized by dominant adhesion forces and high energy dissipation during indentation.

### 2.3. X-Ray Diffraction/X-Ray Fluorescence (XRD/XRF)

**Figure 5:**
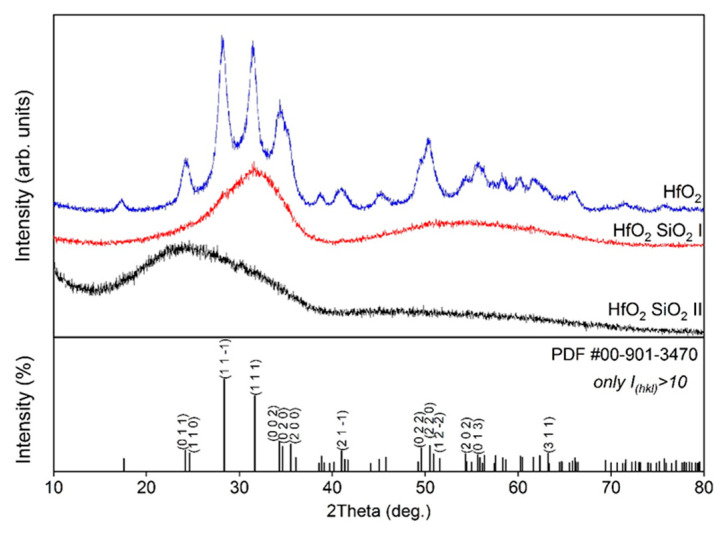
The XRD patterns of the samples HfO_2_, HfO_2_SiO_2_ I, and HfO_2_SiO_2_ II.

The crystal structure and phase evolution of the samples were investigated using X-ray diffraction. The interpretation of the presented X-ray diffraction patterns indicates a major structural difference between the pure sample (sample HfO_2_) and the mixtures containing silica (samples HfO_2_SiO_2_ I and HfO_2_SiO_2_ II). As shown in Figure 5, the diffraction patterns reveal a clear transition from a highly crystalline state to an amorphous one depending on the SiO_2_ content: (i) the sample HfO_2_ exhibits a well-defined crystalline nature. The diffraction lines correspond to a monoclinic single phase (PDF card 00-901-3470) in the P 1 21/c space group. The calculated lattice constants are a = 5.1404(14) Å, b = 5.1639(15) Å, and c = 5.3052(16) Å, with β = 99.055(14); (ii) the sample HfO_2_SiO_2_ I shows a broad halo with small, emerging peaks, indicating an incipient stage of crystallization. Its pattern (middle, red) indicates a predominantly amorphous phase, but with an incipient transition toward a partially crystalline or nanocrystalline state. The amorphous halo is observed between the 20° and 40° 2θ range, with a broad maximum located around 2θ = 30°. Superimposed on this amorphous signal, small peaks can be observed, which correspond to the most intense lines of monoclinic HfO_2_ (such as the (11-1), (111), and (002) planes). The presence of SiO_2_ acts as a crystallization inhibitor. The silica “disrupts” the HfO_2_ lattice, preventing the formation of large crystallites and maintaining the material in an amorphous state, with small crystalline nuclei; (iii) the sample HfO_2_SiO_2_ II displays a purely amorphous character, with no detectable Bragg reflections, quite similar to the SiO_2_ diffraction pattern previously reported [33]. The pattern exhibits only a broad halo between the 15° and 35° 2θ range. The higher concentration of silica leads to the complete amorphization of the sample. According to studies conducted by Wilk et al. [43], the addition of SiO_2_ into the HfO_2_ matrix acts as a stabilizer for the amorphous phase. The shift of the amorphous halo observed in the HfO_2_SiO_2_ I sample compared to the HfO_2_SiO_2_ II sample can be attributed to changes in the short-range order; as the Hf concentration increases, the average interatomic distances decrease, and the electron density rises—a phenomenon documented by Neumayer and Cartier [44] in their analyses of binary oxides. The higher the Hf content, the more compact the amorphous network becomes, exhibiting local atomic arrangements that prefigure the HfO_2_ crystalline lattice.

To better understand the structural differences, XRF analysis was performed to evaluate the elemental distribution. The results, expressed as mass percentages, are summarized in Table 1.

The XRF data strongly corroborate the XRD findings, highlighting the role of SiO_2_ as a crystallization inhibitor. Even a minor addition of Si (0.68% in HfO_2_SiO_2_ I) prevents the full crystallization of the HfO_2_ lattice. When the Si content is increased significantly to 69.49% (HfO_2_SiO_2_ II), the formation of a crystalline lattice is completely suppressed, retaining the amorphous state of the material. This behavior could be correlated with the reported literature data [44,45].

### 2.4. The N_2_ Adsorption–Desorption Measurements

**Figure 6:**
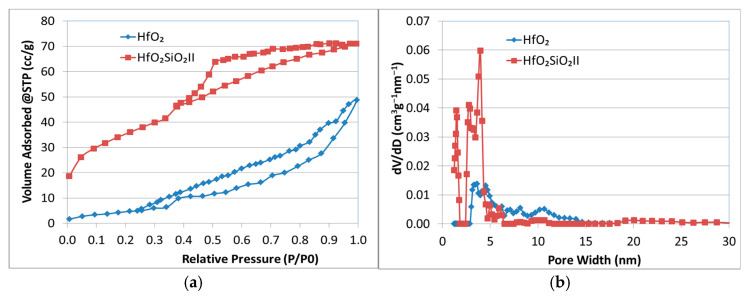
Textural analysis of samples HfO_2_ and HfO_2_SiO_2_ II: (**a**) N_2_ adsorption–desorption isotherms; (**b**) DFT pore size distribution.

The N_2_ adsorption–desorption isotherms and pore size distribution diagrams of the HfO_2_ and HfO_2_SiO_2_ II samples are shown in Figure 6.

Figure 6a shows that the N_2_ adsorption–desorption isotherms of samples HfO_2_ and HfO_2_SiO_2_ II are of type IV according to the IPUAC classification, which is typical of mesoporous materials. While the HfO_2_ sample exhibits an H3-type hysteresis loop, the HfO_2_SiO_2_ II sample shows an H2-type hysteresis loop. The H2 hysteresis type indicates the presence of irregular ink-bottle pores, where large, wide cavities (the “bottle”) are connected to the surface by significantly narrower channels (the “neck”). The HfO_2_ sample exhibits an H3 hysteresis loop, which represents desorption mechanisms that do not denote equilibrium desorption. The H3 type of hysteresis does not show any limiting adsorption as the relative pressure (P/P_0_) approaches 1. This phenomenon is usually attributed to the presence of aggregates of plate-like particles that form slit-shaped pores.

Thus, for the calculation of pore size distribution, we used the density functional theory (DFT) method, which is recommended for more disordered materials, where effects such as pore blocking and cavitation also contribute to hysteresis. DFT methods accurately account for the behavior of fluids in pores and provide much more reliable mesopore size distributions. Figure 6b shows the pore size distribution curves calculated using the DFT method. The recorded curves illustrate polydisperse DFT pore size distributions for both materials, indicating the presence of pores of different sizes, ranging from 3 to 15 nm for the HfO_2_ sample and from 3 to 7 nm for the HfO_2_SiO_2_ II sample. The observed increase in BET surface area (S_BET_) and DFT pore volume for the HfO_2_SiO_2_ II sample, compared with the HfO_2_ sample (Table 2), indicates higher porosity, likely due to the contribution of the SiO_2_ component. Unlike the HfO_2_ sample, the presence of micropores can be observed in the HfO_2_SiO_2_ II sample. Also, the pore widths are narrower for the HfO_2_SiO_2_ II sample.

### 2.5. X-Ray Photoelectron Spectroscopy (XPS)

**Figure 7:**
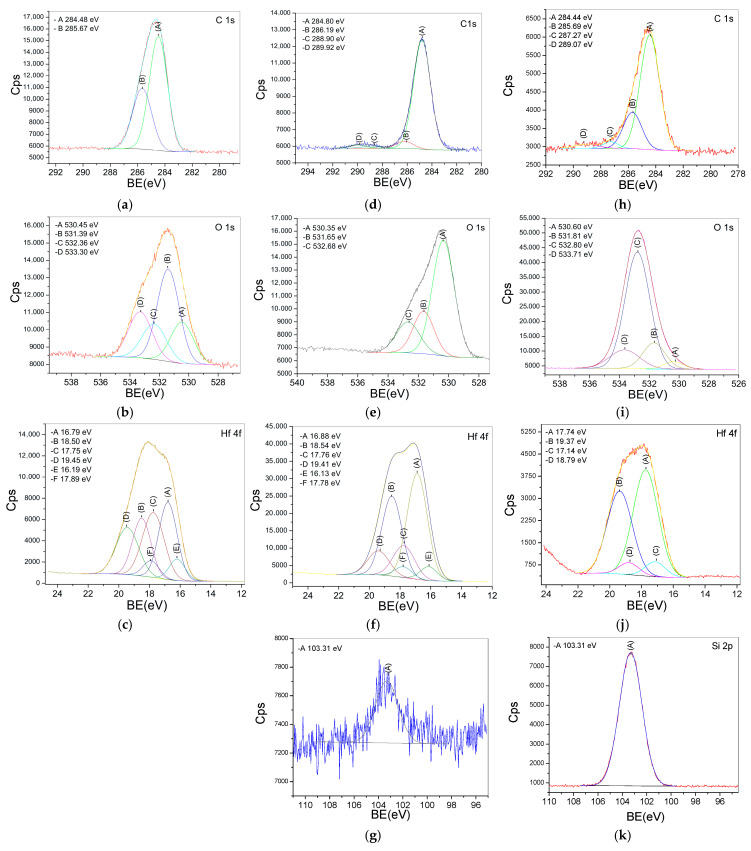
XPS high-resolution C1s, O1s, and Hf4*f* spectra, arranged in columns, recorded for the HfO_2_ (**a**–**c**), HfO_2_SiO_2_ I (**d**–**g**), and HfO_2_SiO_2_ II (**h**–**k**) samples. Si2p spectra were registered for samples HfO_2_SiO_2_ I (**g**) and HfO_2_SiO_2_ II (**k**).

XPS was used to evaluate the surface chemistry of the HfO_2_, HfO_2_SiO_2_ I, and HfO_2_SiO_2_ II samples, confirming the presence of carbon, oxygen, hafnium, and, in the case of HfO_2_SiO_2_ I and HfO_2_SiO_2_ II, silicon. Figure 7a–c show, in the left column, the high-resolution C1s, O1s, and Hf4f spectra of the sample HfO_2_. Deconvolution of the high-resolution C1s spectrum (Figure 7a) resulted in two peaks: peak A, attributed to C-C and C-H single bonds, and peak B, assigned to a C-O single bond [46].

In the O1s spectrum (Figure 7b), peak A was attributed to hafnium oxide [47]; peak B mainly to hafnium hydroxide [47], adsorbed molecular oxygen, or C=O-type bonds; peak C to C-O single bonds; and peak D to hafnium bonds, traces of water, or other carbon-related single bonds. The high resolution of the Hf4f spectrum (Figure 7c) was fitted with a doublet (4f_7/2_ and 4f_5/2_); A-B can be assigned to HfO_2_ [47,48,49], and the C-D doublet can be assigned to the hydroxyl group bonds or Hf(OH)_4_ [49], while the E-F doublet indicates the presence of Hf suboxides [47,48,49].

For the HfO_2_SiO_2_ I sample (Figure 7d–g), the C1s (Figure 7d) spectrum can be deconvoluted into four components. Peak A is assigned to C-C and C-H single bonds, peak B to C-O single bonds, and peak C to O-C=O, -COOH, and -COOR groups, while peak D can indicate the presence of carbonates [46]. The O1s spectrum (Figure 7e) also consists of three contributions. Peak A is attributed to oxygen in hafnium oxide [47], peak B mainly to hafnium hydroxide [47] and adsorbed molecular oxygen or C=O bonds, and peak C to C–O single bonds. In the high-resolution Hf4f spectrum (Figure 7f), the A-B doublet can be assigned to HfO_2_ [47,48,49], the C-D doublet indicates bonds with hydroxyl groups, possibly Hf(OH)_4_ [49], while the E-F doublet indicates Hf suboxides [47,48,49]. The Hf4f peak area ratio of A:C was determined to be 86:14 (%). In the Si2p spectrum (Figure 7g), a single symmetric peak with low intensity is observed, which is assigned to SiO_2_ due to its binding energy.

For the HfO_2_SiO_2_ II sample (Figure 7h–k), the C1s spectrum (Figure 7h) reveals more complex carbon chemistry. Peak A corresponds to C-C and C-H single bonds, peak B to C-O single bonds, peak C to C=O and O-C-O, and peak D to higher oxidation states such as the O-C=O, -COOH, and –COOR groups. The O1s spectrum (Figure 7i) indicates multiple oxygen environments: Peak A is attributed to oxygen in HfO_2_ [47], peak B is attributed mainly to hafnium hydroxide [47], as well as to adsorbed molecular oxygen or carbonyl groups (C=O), and peak C is attributed to Si–O bonds (which become predominant in the spectrum) or C-O single bonds, while peak D is attributed to contributions from hydroxyl groups, adsorbed water, or other single bonds with C. The high-resolution Hf4f spectrum (Figure 7j) presents an A-B doublet attributable to bonds with hydroxyl groups, possibly Hf(OH)_4_ [49], while the C–D doublet indicates HfO_2_. Its binding energy, slightly higher compared with the other samples, may be linked to Hf’s simultaneous bonds with O and -OH [47,48,49]. In the Si2p spectrum (Figure 7k), a single symmetric peak is observed, which is assigned to SiO_2_. Literature data establish correlations between hafnium suboxide formation and the presence of oxygen vacancies [50] and also with the presence of carbon [51].

### 2.6. UV-Vis Spectroscopy

**Figure 8:**
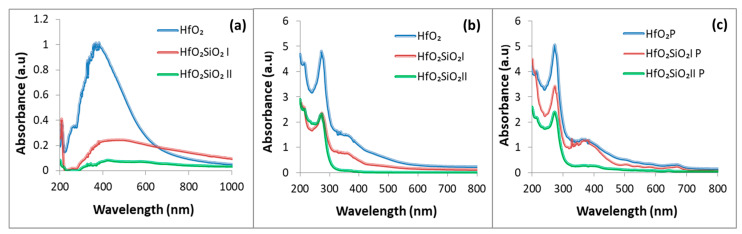
UV-Vis spectra of HfO_2_-based powders (**a**); polyvinyl alcohol (PVA) hydrogels embedding HfO_2_-based powders deposited on glass (**b**) and PVA hydrogels embedding HfO_2_-based powders and porphyrin deposited on glass (**c**).

The UV-Vis spectra presented in Figure 8a show a strong absorption peak for HfO_2_ powder centered at ~380 nm. The high absorption can be assigned to defects and residual surface carbon. Additionally, a noticeable shoulder appears around 280 nm. The spectra of HfO_2_SiO_2_ I and HfO_2_SiO_2_ II samples reveal a decrease in light absorption intensity with increasing SiO_2_ content, accompanied by broader and slightly red-shifted absorption bands. The optical band gap values for the HfO_2_-based samples were obtained based on the Tauc method, assuming indirect electronic transitions. The obtained values are as follows: Eg(HfO_2_) = 5.15 eV; Eg(HfO_2_SiO_2_ I) = 5.55 eV. These experimental results show a small value for pure HfO_2_ compared to the literature data [11] and changes in the electronic band structure induced by adding SiO2 in small amounts to the HfO_2_ matrix, resulting mostly in an increase in the HfO_2_ band gap. In the case of the third sample, which is a composite of HfO_2_ (minor phase) and SiO_2_, the obtained value was Eg (HfO_2_SiO_2_ II) = 5.2 eV. Figure 8b reveals the absorption spectra of HfO_2_-based powders embedded in polyvinyl alcohol (PVA) hydrogels and deposited on glass. The peaks observed between 250 and 300 nm are attributed to the absorption of the glass substrate and the PVA matrix [32].

The peaks in the 300–400 nm range retain the characteristic features of the powders due to the transparency of the polyvinyl alcohol (PVA) matrix, although their intensity is reduced. Upon incorporation of protoporphyrin into the system, the overall light absorption increases, with characteristic porphyrin absorption peaks appearing around 400 nm and in the 500–700 nm range [52].

### 2.7. Zeta Potential Measurements

The modulation of the HfO_2_ surface charge through the incorporation of inorganic (SiO_2_) and organic modifiers, specifically protoporphyrin IX, is illustrated in Figure S1 in the Supplementary Materials. Zeta potential measurements reveal negative values for all samples, with the highest negative surface charge observed for pure HfO_2_ powder (−37 mV), which shifts towards less negative values by SiO_2_ incorporation (the HfO_2_SiO_2_ I, HfO_2_SiO_2_ II samples) and the loading of protoporphyrin IX. This trend is consistent with our previous findings on silica nanotubes, where porphyrin loading similarly raised the zeta potential values of silica [33].

### 2.8. Monitoring of 1,4-Hydroquinone (1,4-H_2_Q) Generation via the Reduction of 1,4-Benzoquinone (1,4-BQ)

**Scheme 1:**
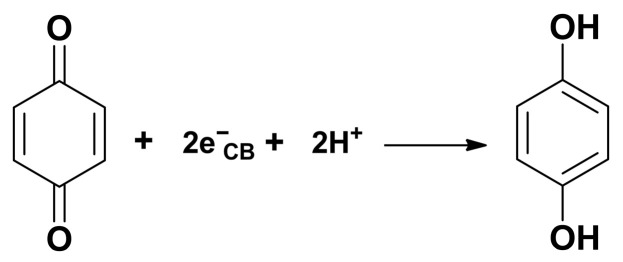
Reaction between 1,4-BQ and photogenerated electrons, leading to 1,4-H_2_Q.

1,4 Benzoquinone (1,4-BQ) is commonly used as a probe molecule to investigate the role of superoxide anion radicals (•O_2−_) [53], as a superoxide radical scavenger in various heterogeneous photocatalytic processes [54,55]. In the presence of oxygen, the reduction of 1,4-BQ to 1,4-hydroquinone (1,4-H_2_Q) competes with the formation of superoxide radicals via electron transfer (O_2_ + e_−_ → O_2−_) as both processes involve photogenerated electrons. To avoid this competition and ensure that the reduction pathway dominates, the photocatalytic measurements were performed under oxygen-deficient conditions, using sealed cuvettes after Ar purging. Under simulated solar irradiation (AM 1.5), the reaction between 1,4-BQ and photogenerated electrons (Scheme 1) at the surface of the investigated samples was monitored in order to comparatively evaluate the amount of 1,4-H_2_Q formed during 5 min of irradiation over each photocatalyst.

**Figure 9:**
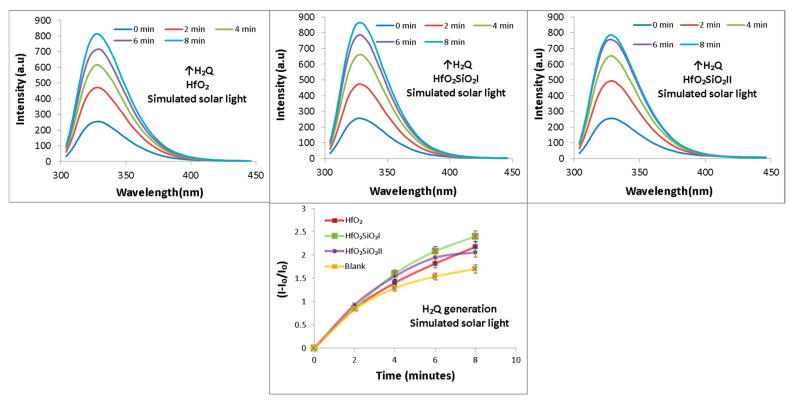
Monitoring of 1,4 hydroquinone photoluminescence signal (λ_ex_ = 288 nm; λ_em_ = 330 nm) under AM 1.5 irradiation and time evolution of its relative amount (I_max_ = 330 nm). The vertical arrows (↑) indicate the increase of H_2_Q amount.

The monitoring of H_2_Q formation presented in Figure 9 shows the time-dependent generation of 1, 4-H_2_Q during 8 min of irradiation under simulated solar light (AM 1.5). Since solar light is a renewable and green energy source, studies involving simulated solar light are particularly valuable for depollution processes. The aim of performing these photocatalytic measurements on hafnium-based materials was mainly to evaluate their ability to photogenerate electrons and transfer them to the 1,4-BQ adsorbed on their surface, reducing it to 1,4-H_2_Q. The photoluminescence signal of 1,4-H_2_Q was monitored every 2 min. Figure 9 shows that, during the first 2 min of irradiation, a similar amount of H_2_Q evolved in all investigated photocatalytic systems, as well as in the blank (BQ solution without catalyst), most likely due to the photolysis of 1,4-BQ, as also reported in the literature [53]. After 6 min of reaction, the HfO_2_SiO_2_ I and HfO_2_ samples show a continuous increase in H_2_Q formation, unlike the HfO_2_SiO_2_ II sample, for which the H_2_Q amount remains almost unchanged.

This behavior may be attributed to the following: (a) catalyst deactivation; (b) the rate of H_2_Q decomposition exceeding its formation rate. Also, it could be related to the material’s ability to promote the formation of superoxide radical anions using the residual molecular oxygen present in the system (Section 2.9). On the other hand, such photocatalytic reactions require a high electron density on the catalyst’s surface. According to literature data, carbon (present as impurity or modifier) can transfer the electrons to the hafnium oxide [51,56], promoting some surface processes. Still, photocatalytic processes carried out on materials with large band gaps such as insulators—and particularly on hafnium oxide—are not fully understood. Most reported studies refer to the engineering of intraband gap defects through impurities (such as residual carbon), added modifiers, nonstoichiometries [18,23,30,31], and surface states capable of photogenerating reactive oxygen species (ROS). The present results demonstrate the ability of HfO_2_-based materials, both unmodified and modified with SiO_2_, to carry out photocatalytic reduction processes under AM 1.5 irradiation.

### 2.9. ROS Photogeneration—Superoxide Radical (•O_2_^−^)

**Figure 10:**
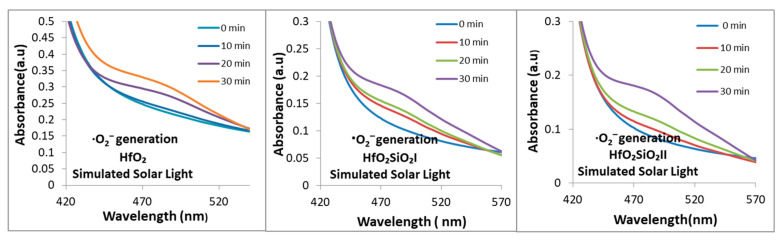
Monitoring the time course of the superoxide radical (•O_2_^–^) generation based on the reduction of aqueous XTT sodium salt to XTT formazan in the presence of HfO_2_, HfO_2_SiO_2_ I, and HfO_2_SiO_2_ II samples under AM 1.5 irradiation.

The generation of the superoxide radical (•O_2_^−^) is presented in Figure 10. According to our previous work [31], XTT formazan (a reddish compound) results from the interaction of the photogenerated superoxide anion radical (•O_2_^−^) formed at the irradiated surface of the investigated powders with XTT sodium salt (100 µM aqueous solution mixed with 2 mg of hafnium-based samples). It is identified by UV-Vis spectroscopy at 475 nm. As shown in Figure 10, all samples are capable of producing •O_2_^−^, with the highest amount observed for the HfO_2_SiO_2_ II sample.

The generation of hydroxyl radical (•OH) was investigated based on our previous work [31], using coumarin as a probe compound. Figure S2 from the Supplementary Materials illustrates the absence of •OH in systems containing hafnium-based photocatalysts.

### 2.10. Monitoring the Generation of Singlet Oxygen (^1^O_2_)

9,10-Dimethylanthracene (DMA) is extensively used as a model compound for monitoring the photogeneration of singlet oxygen (^1^O_2_) by fluorescence measurements [57,58], as it is a well-known singlet oxygen trap [59]. Therefore, upon excitation at λ_ex_ = 370 nm, a decrease in fluorescence emission was observed with increasing singlet oxygen production. Also, the vast majority of the studies involving the photogeneration and valorization of singlet oxygen have been carried out under visible light exposure.

**Figure 11:**
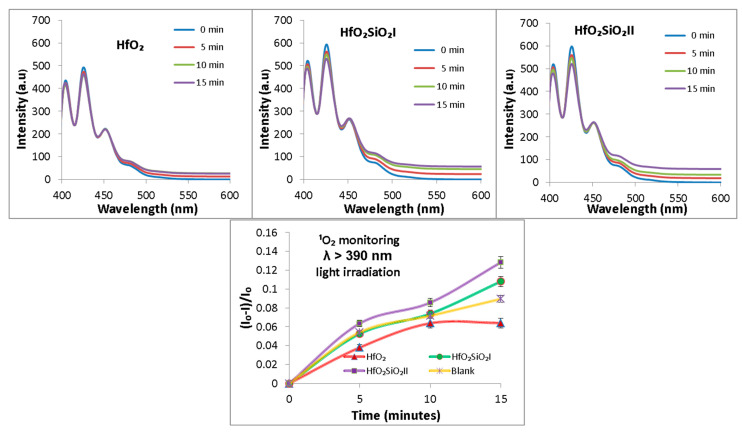
Monitoring the generation of singlet oxygen (^1^O_2_) based on the photoluminescence signal of 9,10-dimethylanthracene (λ_ex_ = 370 nm) in the presence of hafnium oxide-based powders under visible light irradiation (λ > 390 nm).

Figure 11 illustrates that the HfO_2_SiO_2_II and HfO_2_SiO_2_I samples generate singlet oxygen under visible light irradiation (λ > 390 nm). In contrast, the curve corresponding to the bare HfO_2_ sample lies below the blank, indicating that the amount of singlet oxygen is too low to be measured under these conditions. This observation can be explained by its Uv-Vis spectrum, in which the absorption maximum is centered at 380 nm. The highest amount of singlet oxygen was observed for the HfO_2_SiO_2_ II sample, with its formation rate increasing significantly after the first 10 min of irradiation. The amount of singlet oxygen generated by the HfO_2_SiO_2_I sample also becomes measurable after the first 10 min of irradiation; however, its formation rate appears to be significantly slower than that of the former. It can be clearly observed that the amount of singlet oxygen produced by HfO_2_SiO_2_ II and HfO_2_SiO_2_ I under visible light irradiation (λ > 390 nm) depends on the SiO_2_ content and its presence on the catalyst surface. This observation is consistent with our previous work, which demonstrated the ability of the SiO_2_-based materials, obtained by a similar synthesis method, to generate singlet oxygen under visible light exposure [32,33].

### 2.11. DL α-Tocopherol Quenching by the Photogenerated Singlet Oxygen (^1^O_2_)

DL α-tocopherol, a component of vitamin E, is a natural antioxidant and a well-established scavenger of singlet oxygen in biological systems. Numerous studies have employed DL α-tocopherol as a probe molecule to investigate the “in situ” generation of singlet oxygen (^1^O_2_) in both natural and artificial media. This interaction was examined in our previous work by monitoring the reaction between DL α-tocopherol and photogenerated singlet oxygen on SiO_2_ nanotubes modified with iridium compounds and porphyrins under visible light irradiation (λ > 420 nm) [33].

The present study aims to assess the reactivity of singlet oxygen (^1^O_2_), identified using DMA for hafnium-based powders, within three types of photoactive systems:

(a) Hafnium-based powders suspended in a methanolic solution of DL α-tocopherol and exposed to visible light (their optical properties in UV-Vis—Figure 8a).
(b) Films of PVA hydrogels embedding HfO_2_-based powders deposited on glass, immersed in a methanolic solution of DL α-tocopherol and exposed to visible light (their optical properties in UV-Vis—Figure 8b).
(c) Hybrid films of PVA hydrogels embedding protoporphyrin IX-functionalized HfO_2_-based powders/glass immersed in a methanolic solution of DL α-tocopherol and exposed to visible light (λ > 390 nm) (their optical properties in UV-Vis—Figure 8c).

**Figure 12:**
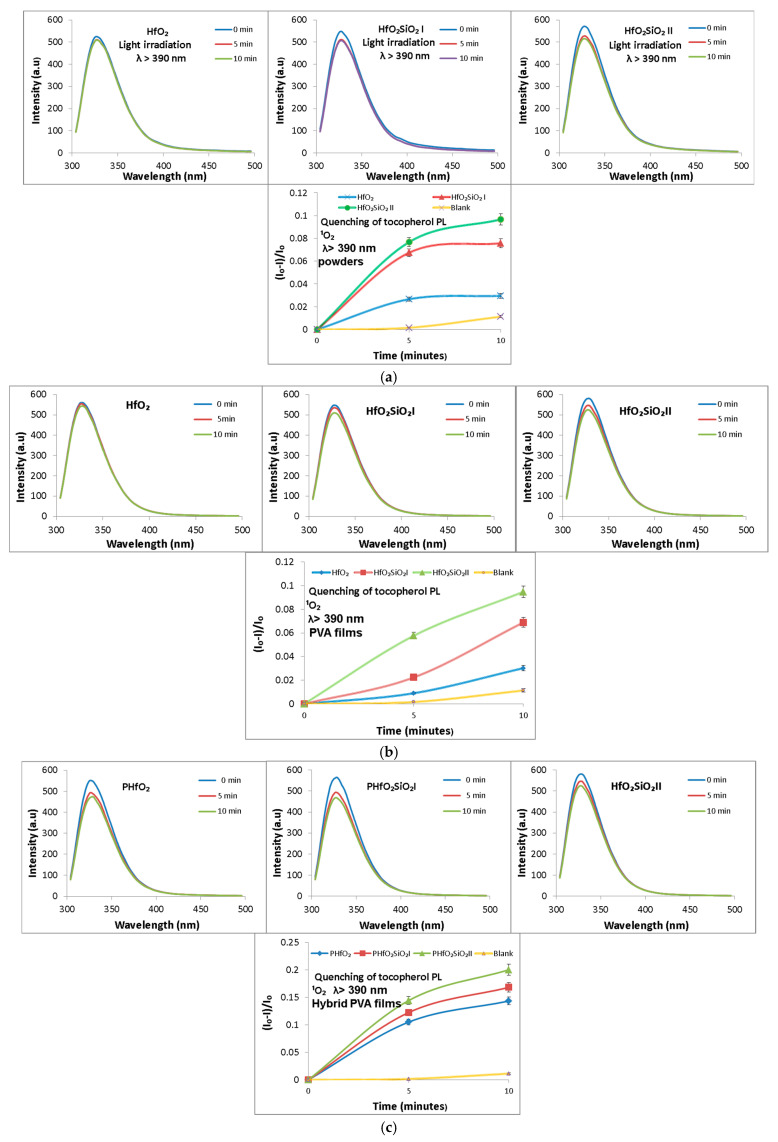
Quenching of DL-α- tocopherol photoluminescence under visible light irradiation (λ > 390 nm) in the presence of HfO_2_-based powders (**a**); quenching of DL-α-tocopherol photoluminescence in the presence of polyvinyl alcohol (PVA) films embedding HfO_2_-based powders (**b**)**;** polyvinyl alcohol (PVA) films embedding HfO_2_-based powders functionalized with protoporphyrin IX (**c**).

The observed decay of DL α-tocopherol photoluminescence in these systems depends not only on the amount of photogenerated singlet oxygen (^1^O_2_) but also on the specific interactions between DL α-tocopherol and the surface of each catalyst. Therefore, it is important to investigate these effects in hafnium-based oxides in different configurations—powders (Figure 12a), films (Figure 12b), and hybrid films (Figure 12c)—with consideration of their potential applications. Furthermore, the incorporation of protoporphyrin IX significantly broadens the range of potential applications by extending activity into the visible region. Protoporphyrin IX is an endogenous compound with well-defined biological functions [60], widely recognized for its ability to generate singlet oxygen and used for its photodynamic properties [61] in cancer therapy. As shown in Figure 12c, the immobilized protoporphyrin IX onto hafnium-based materials embedded in PVA hydrogels enhances light absorption in the visible range. This is expected to promote increased singlet oxygen generation under visible light exposure. The process is monitored by measuring the decrease in DL α-tocopherol photoluminescence intensity, following our previous work [33] and the method reported by Demirkaya-Miloglu et al. [62], using excitation and emission wavelengths of λ_ex_ = 290 nm and λ_em_ = 327 nm, respectively.

Figure 12a shows a decrease in DL α-tocopherol photoluminescence under visible light irradiation (λ > 390 nm), particularly for the HfO_2_SiO_2_ II and HfO_2_SiO_2_ I powders. This overall observation is consistent with Figure 11, which depicts the singlet oxygen produced by the powders and monitored using DMA. Additionally, Figure 12 indicates a high formation rate of singlet oxygen and rapid interactions with DL α-tocopherol during the first 5 min of irradiation for both HfO_2_SiO_2_ II and HfO_2_SiO_2_ I samples (as seen by comparison with Figure 11). According to these results, HfO_2_ also exhibits small activity in singlet oxygen production. Figure 12b demonstrates that, after embedding the hafnium-based powders in PVA and depositing them onto glass as films, the ability of the photoactive materials to generate singlet oxygen is preserved, although it is reduced, especially for the HfO_2_SiO_2_ I sample.

Figure 12c illustrates a significant enhancement in singlet oxygen production for all hybrid PVA films containing hafnium-based materials modified with protoporphyrin IX under visible light irradiation. The activity hierarchy remains the same as in the previous images (Figure 12a,b), emphasizing the preservation of the well-known ability of porphyrin to generate singlet oxygen in the present photoactive systems. Figure S3 illustrates the dark control for quenching DL-α- tocopherol photoluminescence. These findings highlight the potential of such materials for the development of photoactive systems (powders, gels, or coatings) as sources of singlet oxygen, which can be further valorized as antibacterial agents or in photodynamic therapy.

## 3. Conclusions

Hafnium oxide-based nanostructures, synthesized using an adapted sol–gel method, were processed and systematically investigated in bare powder form and embedded in PVA hydrogels deposited onto glass substrates as photoactive films. Three types of materials were obtained and investigated: HfO_2_, HfO_2_ slightly modified with SiO_2_, and a composite in which SiO_2_ is the major phase. XPS characterization identifies the presence of hafnium hydroxides, hafnium oxide, hafnium suboxides, and silica present at the surface of both modified samples. SiO_2_ as the predominant phase induces significant changes in the morphological and textural properties of HfO_2_, as evidenced by STEM, AFM, and BET characterizations. These materials are further functionalized with protoporphyrin IX, for which its presence at the surface of the oxide materials is confirmed by UV-Vis investigation and by zeta potential measurements. Functional characterization of the hafnium oxide-based powders demonstrates their ability to photogenerate reactive oxygen species, specifically superoxide anion radicals under simulated solar light exposure and singlet oxygen upon visible light irradiation (λ > 390 nm). The reactivity of the photogenerated singlet oxygen is confirmed by the quenching of DL α-tocopherol photoluminescence under visible light irradiation (λ > 390 nm) in the presence of HfO_2_-based powders functionalized with protoporphyrin IX and embedded in PVA hydrogels deposited on glass substrates. The evaluation of the mechanical properties of PVA films by AFM, using force–distance spectroscopic measurements and subsequent analysis using the SLS/Zener model, highlighted the viscoelastic behavior of the gels. Hafnium oxide-based powders enable the reduction of 1,4 benzoquinone to 1,4 hidroquinone, demonstrating photocatalytic activity under simulated solar light. Overall, this study provides fundamental evidence for the use of hafnium oxide-based materials as photocatalysts under AM1.5 irradiation and highlights their potential for developing photoactive powders, gels, or films capable of generating singlet oxygen under visible irradiation. Therefore, they show promising applicability as antibacterial agents, as well as in photodynamic therapy as powders, gels, and films. The films exhibit improved recovery and reusability, which favors their potential for technological scale-up (e.g., bio-medical technologies). Therefore, assessment of antibacterial activity and cytotoxic effects on tumor cell lines under light exposure will be investigated in dedicated future work.

## 4. Materials and Methods

### 4.1. Synthesis

#### 4.1.1. Hafnium-Based Powders

Hafnium-based materials were synthesized using an adapted sol–gel method previously employed for the preparation of SiO_2_ nanotubes [30,33], using tartaric acid as an internal template. The precursors used were as follows: hafnium (IV) acetylacetonate (min 96%, Strem Chemicals Inc., Newburyport, MA, USA), tetraethyl orthosilicate (TEOS, 99%, Alfa Aesar, Ward Hill, MA, USA), DL tartaric acid (TA, Riedel de Haen, Seelze, Germany), and absolute ethanol (99.5% Merck, Darmstadt, Germany). The precursor molar ratios were 1.5 Hf(acac)_4_/0.01 TEOS for the HfO_2_SiO_2_ I sample and 1.5 Hf(acac)_4_/1 TEOS for the HfO_2_SiO_2_ II sample. Ammonia gas, bubbled from the heated NH_4_OH solution (30% Roth, Karlsruhe, Germany), was introduced into the ethanolic precursor solution cooled at 0 °C. The resulting compounds were then filtered, dried at 100 °C, and thermally treated in air at 500 °C for 3 h.

#### 4.1.2. Hafnium-Based Powders Functionalized with Porphyrin

A porphyrin stock solution was prepared as follows: 0.005 g of protoporphyrin IX (≥95% Sigma Aldrich, St. Louis, MO, USA) was mixed with 6 mL solution (1:1vol%) of methanol (≥99.8%, Merck, Darmstadt, Germany) and tetrahydrofuran (≥99.9%, Roth, Karlsruhe, Germany). Subsequently, 0.015 g of each pre-synthesized powder was impregnated with 30 µL of the porphyrin-containing stock solution, homogenized, and slowly dried.

Assessments of antibacterial activity and cytotoxic effects on tumor cell lines were planned for future studies. To advance these materials toward biomedical and clinical applications, the protoporphyrin IX-loaded hybrids should be further processed to ensure the complete removal of organic residues (e.g., methanol) and the use of more suitable solvents, such as DMSO instead of tetrahydrofuran.

#### 4.1.3. Films (PVA Hydrogels Embedding HfO_2_-Based Powders Deposited on Glass)

PVA hydrogels were obtained according to previous work [32] using 0.04 g Poly (vinyl alcohol) powder (Sigma-Aldrich, 80% hydrolyzed) and 1 mL of dimethyl sulfoxide (DMSO, Life Technologies, Eugene, OR, USA, MW 78.13). For UV-Vis characterization and DL α-tocopherol quenching measurements, 0.015 g of each powder was mixed with 1 mL of fresh gel aliquots. The resulting mixtures were deposited onto a glass substrate (S = 0.6 × 0.6 cm^2^) and allowed to dry.

#### 4.1.4. Hybrid Films (PVA Hydrogels Embedding Protoporphyrin IX-Functionalized HfO_2_-Based Powders/Glass)

Hybrid films were prepared following the same procedure described above, using hafnium-based powders functionalized with protoporphyrin IX.

### 4.2. Characterization

#### 4.2.1. Scanning Electron Microscopy (SEM)

The morphology of the products was investigated using scanning microscopy (FE-SEM, Thermo Fisher, Waltham, MA, USA, Verios G4 HP). Specimens for surface observations (SEM) were prepared by ultrasonically dispersing powders in deionised water and then depositing a drop of the dispersion onto a polished Al sample holder. To minimize charging effects before the SEM investigation, a 5 nm thick carbon layer was deposited on the specimens. Specimens for STEM-in-SEM investigations were prepared by ultrasonically dispersing the powder samples in methanol, and a drop of the dispersion was deposited onto a lacy carbon film supported on a copper grid. STEM-in-SEM images were captured in bright-field (BF) and high-angle annular dark-field mode (HAADF).

#### 4.2.2. Atomic Force Microscopy (AFM)

Atomic force microscopy (AFM) measurements were performed using an XE-100 microscope from Park Systems (Gwacheon-si, Korea) equipped with flexure-guided cross-talk-eliminated scanners operating in the true non-contact™ mode. AFM images were recorded using NCHR sharp tips (Nanosensors™, Neuchatel, Switzerland) with an ~8 nm tip apex, 125 μm length, 30 μm width, spring constant of ~42 N/m, and 330 kHz resonance frequency. Force–distance spectroscopy measurements were conducted using NSC36A tips (NanoAndMore GmbH, Wetzlar, Germany) with an ~8 nm tip apex, 110 μm length, ~32 μm width, and spring constant of ~1 N/m (90 kHz resonance frequency). Powder samples for AFM imaging were prepared by dispersing HfO_2_-based powders in ultra-pure water, followed by deposition onto clean Si(100) substrates. Hydrogel samples containing HfO_2_-based powders deposited on clean microscopic glass substrates were measured by AFM in the as-prepared state. The recorded AFM images were processed with the XEI program (v 1.8.0—Park Systems) for visualization purposes and are presented in the enhanced contrast mode. Representative line scans are included to illustrate the surface profiles in detail.

#### 4.2.3. X-Ray Diffraction (XRD) Analysis

The X-ray diffraction (XRD) technique was employed to determine the structural nature of the materials (crystalline or amorphous) and to identify the crystalline phases present. XRD measurements were performed with an Ultima IV diffractometer (Rigaku Corp., Tokyo, Japan) using CuKα radiation (λ = 1.5418 Å), operated at 40 kV and 30 mA, and scanning over a range of 2θ = 10–80° at a rate of 2° per minute.

#### 4.2.4. X-Ray Fluorescence (XRF) Analysis

X-ray fluorescence (XRF) spectroscopy was used to determine the elemental composition of materials. The XRF results represent a single-point analysis. The ZSX Primus II wavelength dispersive X-ray fluorescence (WDXRF) spectrometer with ZSX software ver. 5.18 (Rigaku Corp., Tokyo, Japan) was employed. The system also includes Rigaku data analysis software for standardless, semi-quantitative analysis (SQX, ver. 5.18).

#### 4.2.5. N_2_ Adsorption–Desorption Measurements

The experiments were carried out on the calcined samples (500 °C) using a Quantachrome Nova 2200e apparatus (BET surface area, pore volume, and pore size distribution analyzer) produced by Quantachrome Instruments, Boynton Beach, FL, USA. The samples were vacuum-degassed at 400 °C for 3 h prior to the analysis. The textural properties were evaluated using the NovaWin software, ver. 2.1.

#### 4.2.6. Diffuse Reflectance UV–Vis Spectroscopy

Diffuse reflectance UV–Vis spectra of the hafnium-based powders were recorded using a Perkin Elmer Lambda 35 spectrophotometer. The reflectance data were converted into absorption spectra using the Kubelka–Munk function. Diffuse reflectance UV–Vis spectra of the films were also recorded using a PerkinElmer Lambda 35 spectrophotometer (Shelton, CT, USA), employing Spectralon as the reflectance standard. The samples were mounted in a dedicated sample holder and measured over the wavelength range of 200–1000 nm. Due to the very small thickness of the films, the sample holder contributed to the recorded spectra. Therefore, the reflectance of the empty holder (R_cell_) was also measured under identical conditions and used to correct film reflectance (R_film_) according to the relation R_sample_ = (R_film_/R_cell_) × 100 [63].

#### 4.2.7. X-Ray Photoelectron Spectroscopy (XPS) Analysis

X-ray photoelectron spectroscopy (XPS) measurements were performed using a SPECS spectrophotometer (Berlin, Germany) equipped with a PHOIBOS 150 analyzer. The spectra were acquired using a monochromatic radiation source of Specs XR-50M type (Ex = 1486.7 eV) operating at 300 W with a pass energy of 20 eV for the individual spectral lines and 50 eV for the extended spectra. A flood gun (Specs FG15/40 type, SPECS, Berlin, Germany) was employed for charge compensation.

#### 4.2.8. Zeta Potential Measurements

The zeta potential was measured using a Beckman Coulter Delsa Nano C analyzer (Brea, CA, USA) at 25 °C, with all samples dispersed in water as follows: 0.001 g/mL, at pH 7. The measurements were performed in triplicate, and the results are reported as mean values.

#### 4.2.9. Monitoring of 1,4 Hydroquinone Generation from 1,4 Benzoquinone

The photocatalytic reduction of 1,4-benzoquinone to 1,4-hydroquinone was monitored by photoluminescence (PL) spectroscopy. Briefly, 2 mg of powder catalyst was dispersed in 0.5 mL of an aqueous stock solution of 1,4 benzoquinone (1.25 × 10^−4^ M, Sigma Aldrich) and diluted with 3 mL of ultrapure water. The resulting mixture was purged with argon to create an oxygen-deficient atmosphere and sealed in a quartz cuvette. The suspension was then exposed to simulated solar irradiation (AM 1.5, Peccell solar simulator, Japan, PEC-L01, 150 W Short-arc Xe lamp, Asahi Spectra Co., Ltd. Optical Filter, Torrance, CA, USA) and sealed in a cuvette after purging Ar (in an oxygen-deficient atmosphere). The PL signal corresponding to 1,4 hydroquinone formation was recorded after 2, 4, 6, and 8 min. of irradiation using a Carry Eclipse spectrophotometer (Agilent Technologies, Santa Clara, CA, USA) with excitation/emission slit widths set to 5/5. Measurements were performed at an excitation wavelength of λ_ex_ = 288 nm and an emission wavelength of λ_em_ = 330 nm, with excitation and emission slit widths set to 5/5 nm.

#### 4.2.10. Monitoring of Singlet Oxygen (^1^O_2_) Photogeneration

Singlet oxygen (^1^O_2_) generation in the presence of the investigated powders was evaluated by photoluminescence (PL) spectroscopy using a Carry Eclipse fluorescence spectrometer (Agilent Technologies, Santa Clara, CA, USA). The process was monitored by following the decrease in fluorescence intensity of 9,10-dimethylanthracene (99%, Alfa Aesar, Karlsruhe, Germany), used as a chemical probe. Briefly, 0.001 g of each hafnium-based powder was dispersed in a 1 mM DMA solution prepared in methanol (99.9%, AppliChem ITW Reagents, Darmstadt, Germany). The suspensions were irradiated using a solar simulator (Peccel Technologies, Yokohama, Japan, PEC-L01, 150 W Short-arc Xe lamp with a cut-off filter for λ > 390 nm, Asahi Spectra Co., Ltd. Optical Filter, Torrance, CA, USA). The decrease in DMA photoluminescence was recorded at an excitation wavelength of λ_ex_ = 370 nm, using excitation and emission slit widths of 2.5/2.5 nm.

#### 4.2.11. Monitoring of DL α-Tocopherol Quenching

A stock solution of DL alpha-Tocopherol (>96%, Glentham Life Science, Corsham, UK) was prepared from 10 mg DL α-Tocopherol and 5 mL methanol (99.9%, AppliChem ITW Reagents, Darmstadt, Germany). For photoluminescence quenching measurements, a dilute working solution was obtained by mixing 5 mL of the stock solution with 30 mL of methanol. For powder samples, 0.001 g of hafnium oxide-based powders was dispersed in 3.5 mL of the working solution in a quartz cuvette and exposed to visible light irradiation (Peccel solar simulator, PEC-L01 Japan, 150 W Short-arc Xe lamp, cut-off filter for λ > 390 nm, Asahi Spectra Co., Ltd. Optical Filter, Torrance, CA, USA) for 0, 5, and 10 min. For film samples, the films were immersed in the quartz cuvette. The photoluminescence signal of DL α-tocopherol was recorded at an excitation wavelength of λ_ex_ = 290 nm and an emission wavelength of λ_em_ = 327 nm, using excitation and emission slit widths of 2.5/2.5 nm and a scan rate of 120 nm min^−1^.

## Figures and Tables

**Table 1:** Elemental composition of the investigated samples (XRF results).

Sample	Component (*)	Result	Detection Limit	Structural State (XRD)
		Mass %		
HfO_2_	Hf	100	0.16937	Polycrystalline (Monoclinic)
	Si	0	-	
HfO_2_SiO_2_ I	Hf	99.32	0.22603	Amorphous/Nanocrystaline
	Si	0.68	0.05114	
HfO_2_SiO_2_ II	Hf	30.51	0.10085	Amorphous
	Si	69.49	0.09141	

(*): The element lines—Lα for Hf and Kα for Si.

**Table 2:** Textural properties of the analyzed samples.

Sample	S_BET_ (m^2^/g)	V_T_ (cc/g)	V_P_ (cc/g)	D_p_ (nm)	S_DFT_ (m^2^/g)	V_P DFT_ (cc/g)	D_p DFT_ (nm)	S_t_ (m^2^/g)	V_m_ (cc/g)
HfO_2_	19.04	0.075	0.074	4.75	21.24	0.069	3.63	0.000	0.000
HfO_2_SiO_2_ II	123.59	0.110	0.060	3.88	88.33	0.103	3.97	4.047	0.002

V_T_—Total pore volume; V_P_—pore volume, BJH method adsorption branch; D_p_—pore diameter, BJH method adsorption branch; V_P DFT_—pore volume, DFT method; D_p DFT_—pore diameter, DFT method; S_t_—micropore area, thickness method—eBoer; V_m_—micropore volume.

## Data Availability

The original contributions presented in this study are included in the article/supplementary material. Further inquiries can be directed to the corresponding authors.

## Supplementary Materials

The following supporting information can be downloaded at https://www.mdpi.com/article/10.3390/gels12050405/s1. Figure S1—Zeta potential measurements of HfO_2_, HfO_2_SiO_2_ I, HfO_2_SiO_2_ II, and their hybrids with protoporphyrin IX. Figure S2—Monitoring of hydroxyl radical generation under simulated solar irradiation over the investigated catalysts, using P25 TiO_2_ as a reference catalyst. Figure S3—Dark control for quenching of DL-α- tocopherol photoluminescence.

## Abbreviations

The following abbreviations are used in this manuscript:

ROS	Reactive Oxygen Species;
BQ	1,4-benzoquinone;
H_2_Q	1,4-hydroquinone;
SEM	Scanning Electron Microscopy;
AFM	Atomic Force Microscopy;
XRD	X-ray Diffraction;
XRF	X-ray Fluorescence;
XPS	X-ray Photoelectron Spectroscopy;
UV-Vis	Ultraviolet–Visible Spectroscopy;
PL	Photoluminescence.
