# Supplementary material for: Hafnium Oxide-Based Nanostructures as Powders and in Polyvinyl Alcohol Hydrogels for Light-Assisted Processes
Source: Gels. 2026 May 8;12(5):405. doi: 10.3390/gels12050405 (PMC13205915; doi:10.3390/gels12050405)
Supplement: Supplementary file 1 [file gels-12-00405-s001.zip › gels-4251516-supplementary.pdf]

I. Zeta potential measurements

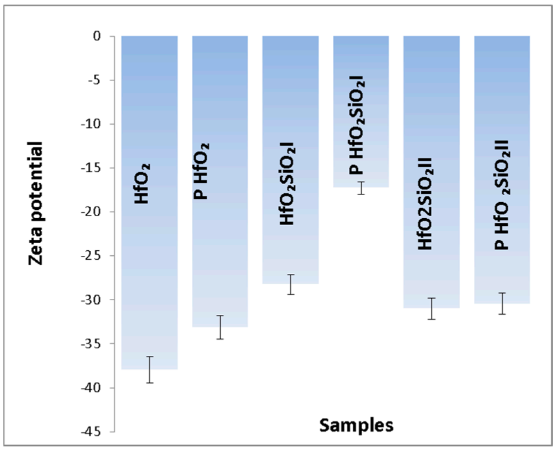

Figure S1. Zeta potential measurements of HfO_2, HfO_2SiO_2 I, HfO_2SiO_2 II, and their hybrids with protoporphyrin IX.

Zeta potential measurements reveal negative values for all samples, with the highest negative surface charge observed for pure HfO_2 powder (- 37.mV). Upon incorporation of SiO_2, the zeta potential shifts towards less negative values, reaching -28 mV for HfO_2SiO_2 I and -31 mV for HfO_2SiO_2 II. The loading of protoporphyrin IX results in an increase in the zeta potential (i.e., a shift toward more positive values) for all HfO_2-based samples. This effect is more pronounced for the $PHfO_2SiO_2$ I sample (-17.2 mV compared to -28.2 mV prior to porphyrin addition). In contrast, only a minor shift is observed for HfO_2SiO_2 II after porphyrin immobilization (-30.4 mV compared to -31 mV for the unmodified sample).

II. Monitoring of hydroxyl radical (•OH) generation using coumarin as a probe molecule

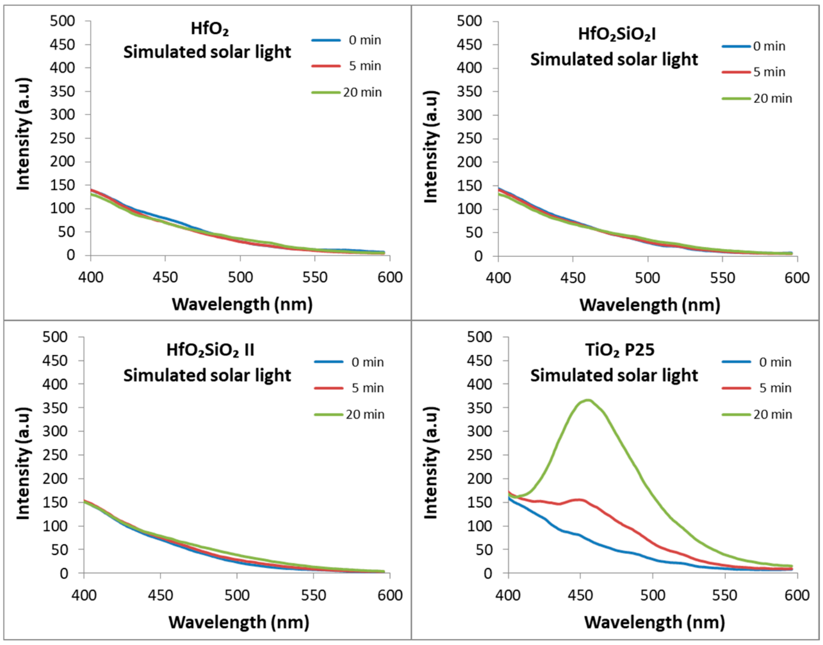

Figure S2. Monitoring of hydroxyl radical generation under simulated solar irradiation over the investigated catalysts using P25 TiO_2 as a reference catalyst.

The photoluminescence signal at 451 nm (λ_{ex} =330 nm; λ_{em} = 451 nm) is characteristic of the formation of the photoluminescent product umbelliferone, which results from the reaction between coumarin and hydroxyl radicals photogenerated by the catalysts under AM 1.5 irradiation. Therefore, the time-dependent formation of umbelliferone can be directly correlated with hydroxyl radical (•OH) generation.

As shown in Figure S2, the hafnium-based photocatalyst did not generate detectable hydroxyl radicals under the applied experimental conditions. For comparison, hydroxyl radical generation was also monitored under identical conditions in the presence of P25 TiO_2 (Degussa), which was used as a reference photocatalyst. In this case, a strong emission peak centered at 451 nm was observed, indicating substantial •OH generation, consistent with our previously reported data [1].

III. Dark control for quenching of DL-α- tocopherol photoluminescence

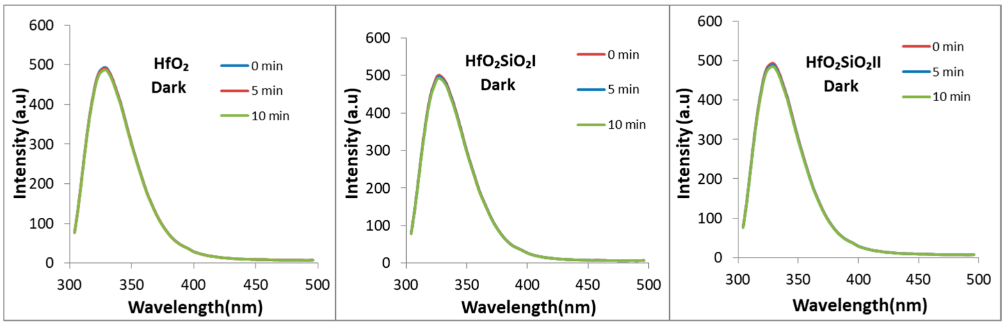

Figure S3. Dark control for quenching of DL-α- tocopherol photoluminescence.

According to Figure S3, no significant quenching of the Tocopherol PL was registered.

References

1. Anastasescu, C.; Negrila, C.; Angelescu, D.G.; Atkinson, I.; Anastasescu, M.; Spataru, N.; Zaharescu, M.; Balint, I. Distinct and interrelated facets bound to photocatalysis and ROS generation on insulators and semiconductors: Cases of SiO_2, TiO_2 and their composite SiO_2-TiO_2. *Catal. Sci. Technol.* **2018**, *8*, 5657–5668
